# Prevalence of pulmonary tuberculosis among the tribal populations in India

**DOI:** 10.1371/journal.pone.0251519

**Published:** 2021-06-04

**Authors:** Beena E. Thomas, Kannan Thiruvengadam, Chandrasekaran Vedhachalam, Srividya A, V. G. Rao, Paluru Vijayachari, Yadav Rajiv, Raghavi V, Avi Kumar Bansal, Anil Kumar Indira Krishna, Alex Joseph, Anil Purty J, Tahziba Hussain, Praveen Anand, Pradeep Das, K. R. John, Rekha Devi K., Sunish P, Azhagendran S, Azger Dusthakeer, Bhat J, Vineet K. Chadha, Toteja G. S., Dasarathy Raghunath, Madhuchhanda Das, A. M. Khan, Hapreet Kaur

**Affiliations:** 1 Department of Social and Behavioral Research, ICMR – National Institute for Research in Tuberculosis, Chennai, India; 2 Department of Statistics, Epidemiology Unit, ICMR – National Institute for Research in Tuberculosis, Chennai, India; 3 Department of Biostatistics, ICMR – Vector Control Research Centre, Pondicherry, India; 4 Division of Communicable Diseases, ICMR – National Institute for Research in Tribal Health, Jabalpur, India; 5 ICMR – Regional Medical Research Centre, Port Blair, Andaman and Nicobar Islands; 6 Department of Epidemiology, ICMR – National JALMA Institute for Leprosy & Other Mycobacterial Diseases, Agra, India; 7 School of Public Health, SRM Institute of Science and Technology, Kattankulathur, Tamil Nadu, India; 8 Department of Community Medicine, Pondicherry Institute of Medical Sciences, Pondicherry, India; 9 ICMR – Regional Medical Research Centre, Bhuvaneshwar, India; 10 Department of Epidemiology, ICMR – Desert Medicine Research Centre, Jodhpur, India; 11 Rajendra Memorial Research Institute of Medical Sciences, Patna, India; 12 Department of Community Medicine, Apollo Institute of Medical Sciences & Research, Chittoor, India; 13 ICMR – Regional Medical Research Centre, Dibrugarh, India; 14 Department of Bacteriology, ICMR – National Institute for Research in Tuberculosis, Chennai, India; 15 Central Leprosy Teaching and Training Institute, Chengalpet, India; 16 Tribal Task Force, ICMR – Former Dean, Armed Forces Medical College, Pune, India; 17 Division of Communicable Diseases (ECD), Indian Council of Medical Research, New Delhi, India; The Foundation for Medical Research, INDIA

## Abstract

**Importance:**

There is no concrete evidence on the burden of TB among the tribal populations across India except for few studies mainly conducted in Central India with a pooled estimation of 703/100,000 with a high degree of heterogeneity.

**Objective:**

To estimate the prevalence of TB among the tribal populations in India.

**Design, participants, setting:**

A survey using a multistage cluster sampling design was conducted between April 2015 and March 2020 covering 88 villages (clusters) from districts with over 70% tribal majority populations in 17 States across 6 zones of India. The sample populations included individuals ≥15 years old.

**Main outcome and measures:**

Eligible participants who were screened through an interview for symptoms suggestive of pulmonary TB (PTB); Two sputum specimens were examined by smear and culture. Prevalence was estimated after multiple imputations for non-coverage and a correction factor of 1.31 was then applied to account for non-inclusion of X-ray screening.

**Results:**

A total of 74532 (81.0%) of the 92038 eligible individuals were screened; 2675 (3.6%) were found to have TB symptoms or h/o ATT. The overall prevalence of PTB was 432 per 100,000 populations. The PTB prevalence per 100,000 populations was highest 625 [95% CI: 496–754] in the central zone and least 153 [95% CI: 24–281] in the west zone. Among the 17 states that were covered in this study, Odisha recorded the highest prevalence of 803 [95% CI: 504–1101] and Jammu and Kashmir the lowest 127 [95% CI: 0–310] per 100,000 populations. Findings from multiple logistic regression analysis reflected that those aged 35 years and above, with BMI <18.5 Kgs /m^2^, h/o ATT, smoking, and/or consuming alcohol had a higher risk of bacteriologically positive PTB. Weight loss was relatively more important symptom associated with tuberculosis among this tribal populations followed by night sweats, blood in sputum, and fever.

**Conclusion and relevance:**

The overall prevalence of PTB among tribal groups is higher than the general populations with a wide variation of prevalence of PTB among the tribal groups at zone and state levels. These findings call for strengthening of the TB control efforts in tribal areas to reduce TB prevalence through tribal community/site-specific intervention programs.

## Introduction

India continues to have the highest Tuberculosis (TB) burden in the world as it accounts more than one fourth (27%) of the globally TB reported cases [1]. Though the Government of India (GoI) has taken several steps towards TB elimination, the disease continues to be a major public health problem in the country. After a nationwide disease survey conducted during 1955–1958 on the TB disease situation in the general populations of the country for the first time [2], there have been studies reporting nationwide estimates of PTB with a high degree of variation. A recent pooled estimate of the prevalence of bacteriologically positive Pulmonary Tuberculosis (PTB) in India was estimated as 296 per 100,000 populations [3]. The limitation of this estimate was the heterogeneity of the populationss across the studies and lack of correction factor for non-inclusion of X-ray. A meta-analysis from 5 different large scale prevalence studies, accounting for X-ray screening provided an overall pooled estimate of 350 (95% CI: 261–439) per 100,000 populations [4]. While we have these different estimates to understand the prevalence of TB among the general populations, some of which include the tribal populations in their estimates, we do not have enough data exclusively on the prevalence of TB among the tribal populations that constitutes 8.6 per cent of India’s populations [5].

While tribal groups are diverse they share certain characteristics such as poor health indicators, a greater burden of morbidity and mortality, and very limited access to healthcare services [6]. Although TB is a major health problem among tribal communities studies conducted on this populations are rather limited mostly to central India [7–12]. A pooled estimate of the PTB prevalence among the tribal populations was reported as 703/100,000 [13]. The limitation with this finding is that it is based on a few studies with a high degree of variation [7, 8, 11, 12, 14–16] in the PTB prevalence ranging from 146 per 100,000 among the Baiga tribe to 1995 per 100,000 among the Saharia tribe [14, 17].

These findings emphasized the need to estimate the prevalence of TB among this populations across India which is an important epidemiological indicator of the TB burden among the tribal populations [15, 18] It is against this background that this study was carried out to understand the burden of PTB among the tribal populations.

## Materials and methods

### Sample size and sampling design

The survey was conducted between April 2015 and March 2020 among individuals (tribes) aged ≥15 years in tribal villages (clusters) selected based on populations proportional to the estimated size (PPES) method. A sample size of 92038 was estimated by assuming a disease prevalence of 387/100,000 populations [16] precision of 15%, a design effect of 1.3 [19] at 5% level of significance, and a non-response of 10%.

A multistage cluster sampling design without replacement was adopted. The entire country was divided into 6 zones, each with two or more states: East, West, North, South, Central, and North East (S1 Map). In each zone, districts (with >70% tribal majority populations) were listed along with the list of villages in these tribal districts. Once this list was complete, the villages (clusters) from these districts were selected based on PPES (S2 Map). A total of 88 villages were selected from 17 states of India. To achieve the sample size, a minimum of 800 individuals were required from each selected village where the streets were randomly selected to cover the required sample size. If the selected villages/ clusters were smaller and could not cover this required sample, an adjacent village from those listed was selected to cover the number of individuals required.

### TB survey

The field investigators for the study were carefully selected across the sites and care was taken to select those who could speak the local language and were from the districts selected from the study. They underwent intense training which included how to approach tribal populationss, screen for symptoms, and most importantly on how to collect quality sputum storage and transportation of sputum.

Prior to the survey, planning visits to the districts and each of the villages was conducted with the study principal investigators and the team to meet the district officials and TB program personnel for their approvals for the study. This was followed by visits to the tribal villages to meet with the influential people in the village heads of the villages, panchayat leaders, representatives from tribal youth, men, and women. Through these individual and group meetings, the purpose of the study was explained and communities prepared for the survey and interviews which were sensitive as it included identifying individuals with presumptive TB, collecting information on risk factors such as smoking and alcohol use, and sputum collection. This important exercise was further facilitated by using community participatory approaches that involved social mapping to understand the demographics and lifestyle of the people in the village and to establish a rapport with the community to gain their trust before initiation of the survey.

All the households in the selected villages were line listed and registered by door-to-door visits for an interview to elicit information on individuals 15 years or older with symptoms suggestive of PTB. The. symptoms included persistent cough with and without expectoration for more 2 weeks or more; chest pain for 1 month or more; fever for ≥1 month; loss of weight, night sweats, and haemoptysis anytime over the last 6 months. Individuals with any one or more of these symptoms (‘*chest symptomatic persons*’) as well as those currently on anti-TB treatment were considered eligible for collection of sputum. For those with symptoms, two sputum samples were collected in falcon tubes: the first on the spot and the second in the morning of the next day. The samples marked with UID of the individuals were transported under the cold chain to the nearest Revised National Tuberculosis Control Program (RNTCP) Intermediate Reference Laboratory (IRL) laboratory on the same day where possible and in difficult terrains within 72 hours for smear and culture investigations as per RNTCP guidelines [20] Those individuals, who were not available for examination on the day of the visit, were revisited and three attempts were made to ensure at least 90% of the study populations had undergone investigations. Data included social and demographic details, history of anti-TB treatment (ATT), alcohol consumption, tobacco smoking, height, and weight of individuals.

### Definition of a PTB bacteriologically positive case

Any individual with one or both sputum specimen bacteriologically positive (positive for AFB on microscopy and/or for *M*. *tuberculosis* on culture) was considered positive for PTB.

### Data management

The study data were directly entered into the structured electronic form with the validation and logical constraints, developed using Open Data Kit (ODK) (opendatakit.org), an open-access software tool. During the field operation, data collected was uploaded directly to the main server and reviewed for quality assurance. Survey data were analyzed using IBM SPSS Statistics for Windows, Version 25.0. Armonk, NY: IBM Corp. Estimated prevalence were presented as map, created using ArcGIS version 10.7.1 (Licenced to our institute ICMR—National Institute for Research in Tuberculosis, Chennai, India) with the help of geo referenced data.

### Statistical methods

#### TB prevalence based on multiple imputations for missing observations

In this study, we report the bacteriologically positive PTB prevalence estimated using the multiple imputation (MI) technique [21] accounting for the missing observations as described in the following paragraph.

To correct for bias introduced by the incompleteness of data in the estimation of PTB prevalence, multiple missing value imputation was done for noncoverage and nonresponse Twenty imputed data sets were created. An individual level analysis using a logistic regression model with robust standard errors was done with and without imputation. Subsequently, cluster level analysis was performed using random effects (household level effects) logistic regression model with and without imputation to estimate the TB prevalence based on the pooled dataset. This model accounts for both clustering effect due to the sampling design and the uncertainty introduced by imputation of missing values while estimating the 95% CI (Confidence Intervals) for the prevalence of PTB.

The prevalence obtained through multiple imputations was then adjusted for X-ray screening with a correction factor of 1.31 [4] considered as the final estimate of the prevalence of bacteriologically positive PTB: overall, zone wise, state wise, gender, and age-specific. Chi-square test with continuity correction and Chi-square test for linear trend was used to test the significance of differences between proportions and linearity of PTB prevalence by age group, respectively. Relative importance of chest symptoms in predicting the presence of tuberculosis was determined by the general dominance index. Univariate and multiple logistic regression analyses were performed for associations between PTB and risk factors; age, gender, BMI, smoking, alcohol consumption, and history of ATT. (P-value <0.05 were considered statistically significant).

#### Approval and ethical clearance for the study

The ethical clearance was obtained from the Institutional Ethics Committees (IEC) of ICMR—National Institute for Research in Tuberculosis, Chennai, India; ICMR—National Institute for Research in Tribal Health, Jabalpur, India; ICMR—Regional Medical research center, Port Blair, India; ICMR—National JALMA Institute for Leprosy & Other Mycobacterial Diseases, Agra, India; SRM Medical College Hospital & Research Centre, Chennai, India; Pondicherry Institute for Medical Sciences, Pondicherry, India; ICMR—Regional Medical Research Centre, Bhuvaneshwar, India; ICMR—Desert Medicine Research Centre, Jodhpur, India; ICMR—Rajendra Memorial Research Institute of Medical Sciences, Patna, India and ICMR—Regional Medical Research Centre, Dibrugarh, India; before initiation of the project. Approval was also obtained from the Tribal Commission/tribal welfare board and the Local tribal leaders from each site. Eligible participants were explained in detail about the study through a participants information sheet for uniformity. Written consent of those willing was obtained before enrolment to the study.

## Results

**Fig 1:**
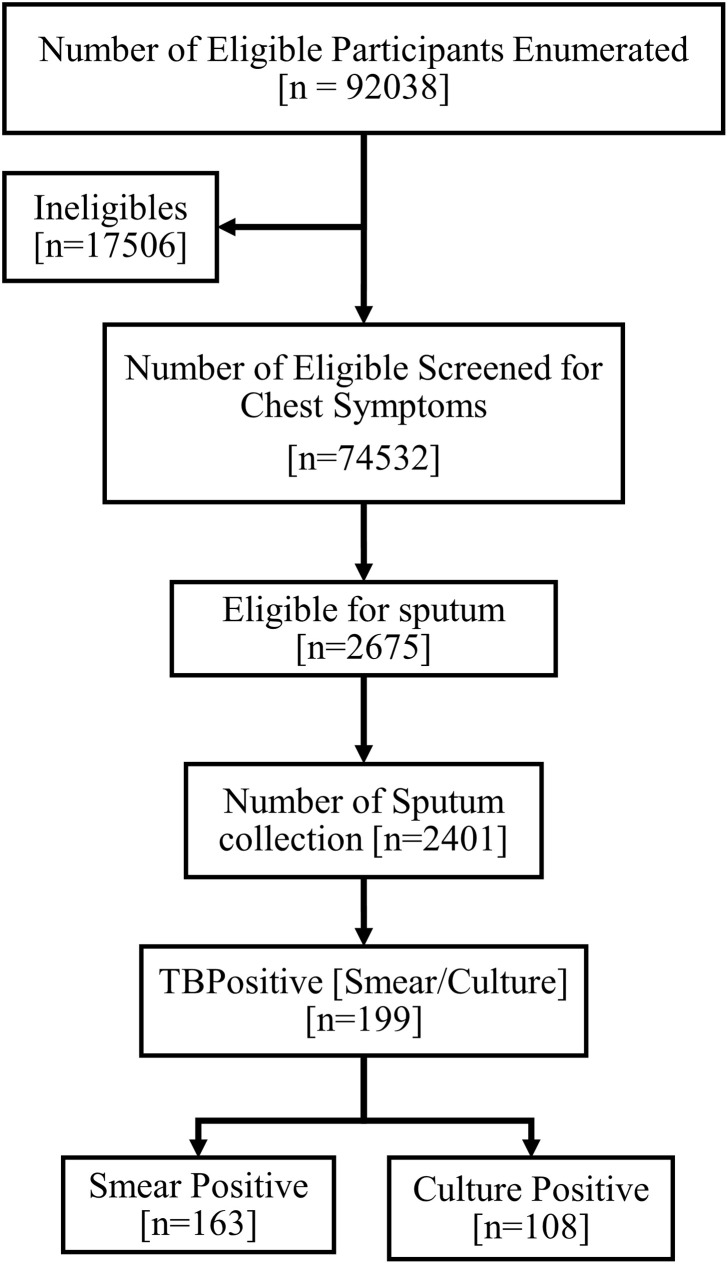
Study populations.

A total of 92,038 eligible individuals (≥15 years of age) were enumerated across 88 villages of India. Out of this, 74,532 (81.0%) were screened for symptoms suggestive of TB, and 2,675 (3.6%) were found to have at least one of the TB symptoms (Fig 1). Of them, 768 (1.0%) had previously undergone ATT, 142 (0.2%) were currently on ATTand 2,218 (3.0%) had symptoms suggestive of TB.

Of the 2,675 people eligible for sputum examination, 2,401 (89.8%) produced sputum samples of which 163 (6.8%) were smear positive, 108 (4.5%) culture positive and 199 (8.3%) bacteriologically positive.

### Prevalence is based on multiple imputation (MI) and correction factors for non-inclusion of X-ray (Tables 1 and 2)

**Table 1:** Prevalence of smear and/or culture positive pulmonary tuberculosis by zone (per 100,000 populations).

Zone	Eligible	Screened (%)	Sputum Eligible (%)	Sputum Collected (%)	Prevalence Per Lakh (95% CI)	Adjusted for Chest X-ray (95% CI)
**Centre**	22765	18760 (82.4)	913 (4.9)	889 (97.4)	477 (379–575)	625 (496–754)
**East**	16760	15246 (91.0)	446 (2.9)	391 (87.7)	327 (224–431)	429 (293–565)
**North**	18703	13632 (72.9)	581 (4.3)	509 (87.6)	320 (224–415)	419 (294–544)
**North East**	14461	11574 (80.0)	309 (2.7)	271 (87.7)	233 (134–331)	305 (176–433)
**South**	10780	8422 (78.1)	282 (3.3)	198 (70.2)	341 (200–481)	446 (262–630)
**West**	8569	6898 (80.5)	144 (2.1)	143 (99.3)	117 (19–215)	153 (24–281)
**Overall**	92038	74532 (81.0)	2675 (3.6)	2401 (89.8)	330 (285–375)	432 (373–491)

X-ray Correction factor—1.31

**Table 2:** Prevalence of smear and/or culture positive pulmonary tuberculosis by state wise (per 100,000 populations).

State wise	Eligible	Screened (%)	Sputum Eligible (%)	Sputum Collected (%)	Prevalence Per Lakh (95% CI)	Adjusted for Chest X-ray (95% CI)
**Andaman & Nicobar**	3806	3302 (86.8)	96 (2.9)	82 (85.4)	495 (243–748)	649 (318–979)
**Andhra Pradesh**	4127	2568 (62.2)	88 (3.4)	59 (67.0)	286 (53–518)	374 (69–679)
**Bihar**	3409	2809 (82.4)	54 (1.9)	53 (98.1)	177 (0–355)	232 (0–465)
**Chhattisgarh**	6700	5126 (76.5)	283 (5.5)	278 (98.2)	420 (246–594)	550 (322–778)
**Himachal Pradesh**	3171	2433 (76.7)	157 (6.5)	147 (93.6)	329 (96–562)	431 (126–736)
**Jammu & Kashmir**	2917	2347 (80.5)	43 (1.8)	43 (100)	97 (0–237)	127 (0–310)
**Jharkhand**	7782	7292 (93.7)	244 (3.3)	216 (88.5)	189 (64–314)	248 (84–412)
**Madhya Pradesh**	16065	13634 (84.9)	630 (4.6)	611 (97.0)	501 (380–622)	656 (498–815)
**Maharashtra**	8569	6898 (80.5)	144 (2.1)	143 (99.3)	117 (19–215)	153 (24–281)
**Manipur**	3931	2473 (62.9)	84 (3.4)	78 (92.9)	274 (70–477)	358 (92–625)
**Meghalaya**	3628	3266 (90.0)	73 (2.2)	58 (79.5)	139 (0–300)	182 (0–392)
**Nagaland**	3539	3380(95.5)	82 (2.4)	65 (79.3)	304 (95–512)	398 (125–671)
**Odisha**	5569	5145(92.4)	148 (2.9)	122 (82.4)	613 (385–841)	803 (504–1101)
**Rajasthan**	9262	6482 (70)	294 (4.5)	232 (78.9)	445 (291–598)	582 (382–783)
**Tamil Nadu**	2847	2552 (89.6)	98 (3.8)	57 (58.2)	214 (0–459)	280 (0–602)
**Tripura**	3363	2455 (73)	70 (2.9)	70 (100)	210 (21–399)	275 (27–523)
**Uttar Pradesh**	3353	2370 (70.7)	87 (3.7)	87 (100)	160 (0–345)	210 (0–453)
**Overall**	92038	74532 (81.0)	2675 (3.6)	2401(89.8)	330 (285–375)	432 (373–491)

X-ray Correction factor—1.31

The overall prevalence of smear positive, culture positive, and bacteriologically positive PTB following MI and corrected for X-ray screening were estimated to be 357 (95% CI 301–414), 234 (95% CI 188–280), and 432 (95% CI: 373–491) per 100,000 populations, respectively.

The PTB prevalence per 100,000 populations was highest 625 [95% CI: 496–754] in the central zone and least 153 [95% CI: 24–281] in the west zone (S1 Map). Among the 17 states, Odisha recorded the highest prevalence of 803 [95% CI: 504–1101] and Jammu and Kashmir the lowest 127 [95% CI: 0–310] per 100,000 populations (S2 Map).

### Trends of PTB prevalence: Age and gender

**Fig 2:**
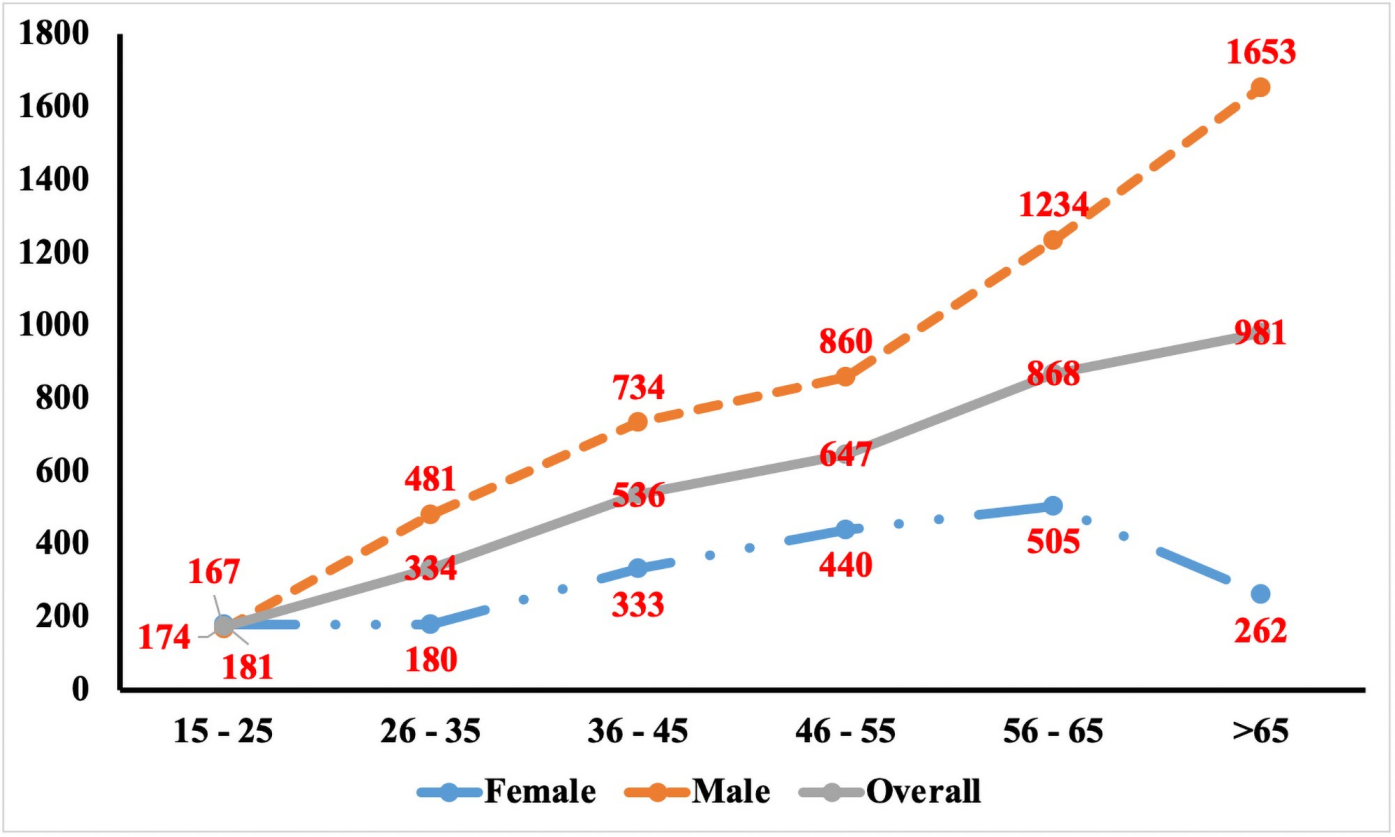
Age and gender wise prevalence of bacteriologically positive pulmonary TB based on MI corrected for Xray screening.

It was observed that PTB prevalence increased with age with 868 (95% CI 609–1128) and 981 (95%CI 606–1356) in the age group of 56–65 years and >65 years respectively. Prevalence was significantly higher among males across all age groups (Fig 2).

### Relative importance of chest symptoms

Among the chest symptoms, the most common symptoms reported were cough (90.2%), chest pain (72.2%), cough with expectoration (63.9%), and weight loss (40.6%). General dominance analysis pointed to weight loss relatively being the prominent symptom in the prediction of PTB followed by night sweat, blood in sputum, and fever. (S1 Fig).

### Factors associated with PTB

With Multiple logistic regression analysis, the predominant factors associated with TB were >aged 35years, BMI <18.5 Kgs /m^2^, history of ATT, smoking and/or consuming alcohol adjusting for cluster effects and covariates of gender, smoking, alcohol, age, occupation, anti-TB treatment and BMI. (Table 3 and S1 Table).

**Table 3:** Multivariate analysis for the factors associated with the occurrence of pulmonary tuberculosis.

	Smear Positive	pValue	Culture Positive	pValue	Smear and/or Culture Positive	pValue
	aOR (95% CI)		aOR (95% CI)		aOR (95% CI)	
**Age Group**						
15–34	1.00		1.00		1.00	
35–54	2.39 (1.45–3.95)	<0.001	3.17 (1.83–5.48)	<0.001	2.66 (1.71–4.13)	<0.001
≥55	3.20 (1.81–5.68)	<0.001	3.15 (1.60–6.19)	0.001	3.47 (2.01–5.97)	<0.001
**Gender**						
Female	1.00		1.00		1.00	
Male	2.11 (1.48–3.01)	<0.001	1.88 (1.15–3.06)	0.012	2.16 (1.60–2.91)	<0.001
**Occupation**						
Unemployed	1.00		1.00		1.00	
Employed	2.6 (0.79–8.56)	0.114	1.51 (0.27–8.39)	0.631	1.78 (0.55–5.82)	0.335
**Smoking and Alcohol consumption**						
No	1.00		1.00		1.00	
Alcohol only	0.40 (0.22–0.73)	0.003	0.34 (0.12–0.91)	0.033	0.38 (0.22–0.66)	<0.001
Smoking Only	1.50 (0.77–2.92)	0.233	2.24 (0.89–5.59)	0.084	1.45 (0.71–2.99)	0.308
Both	1.69 (1.07–2.69)	0.026	1.95 (0.99–3.85)	0.054	1.66 (1.11–2.47)	0.014
**Previous treated with ATT**						
No	1.00		1.00		1.00	
Yes	10.62 (6.36–17.72)	<0.001	13.39 (7.33–24.45)	<0.001	10.24 (6.28–16.71)	<0.001
**Body Mass Index**						
≥18.5	1.00		1.00		1.00	
<18.5	3.33 (2.16–5.13)	<0.001	3.20 (2.05–5.01)	<0.001	2.99 (1.99–4.49)	<0.001

aOR—Odds Ratio; aOR was estimated after adjusting for the covariates and cluster effects.

It may be observed from the multiple analysis that there is an age dependent increase in PTB prevalence comparing the risk of PTB in those ≥35 and <35 years. Those between 35-54years had 2.71 (95% CI: 1.73–4.22) times higher risk than those <35 years with a clearer difference between those ≥ 55 years who had 3.51(95% CI: 2.06–5.99) times higher risk than those <35 years. With regard to gender, males irrespective of age had 2.14 (95% CI: 1.60–2.87) times higher risk compared to females (Fig 2). While the risk for PTB increased by 1.57 (95% CI: 1.04–2.37) with alcohol use and smoking, a low BMI of <18.5 had an increased risk of 2.91 (95% CI: 1.96–4.33) times and a 10.44 (95% CI: 6.45–16.91) times higher risk for those who had a history of TB.

## Discussion

This is the first study of its kind that reports the prevalence of pulmonary TB among the tribal populations across India. The study gains importance as the only reference to the overall prevalence of TB among the tribal populations till date was based on a meta-analysis by Thomas et al., 2015 [13] that reported a pooled PTB prevalence estimate of 703 (95%CI: 386–1011) per 100000 with a high degree of variation indicated by the wide confidence intervals. The results of the current study report an overall prevalence of PTB as 432 per 100000 (95% CI: 373–491), with a much smaller confidence interval. The high estimate in the meta-analysis could have been due to the fact that 5 of the 7 studies, were from Madhya Pradesh in Central India [7, 12, 14, 16, 22], and among them, 3 were on Sahariya tribes with the prevalence of PTB reported between of 387–1518 per 100,000 populations [7, 15, 16].

The bacteriologically positive PTB prevalence (432/100,000) among tribal populationss identified from our findings is higher compared with the PTB prevalence in the general populations based on the pooled estimate of 350 (95%CI: 261–439) per 100,000 [4]. While we have chosen to compare our findings with this estimate as the methods employed are similar, there are two more studies that have reported pooled estimates of 296 and 316 per 100,000 [3, 23].

Our study has highlighted the heterogeneous distribution of PTB among the tribal populations within zones, states, and districts. Although the sample sizes at the zone and state level in the current study may not have enough power to estimate the PTB prevalence at those levels, the results provide insight into the variability of PTB across the zones and states. Our findings point to a higher prevalence of PTB in the central zone. These observations corroborate with the results of studies carried out in central India which reported a high TB prevalence in the tribal populationss in the different districts [7, 12, 15, 24]. The reasons for the high prevalence of PTB among the central India tribal communities have been attributed to various social and structural factors which included poverty, housing, access to health care facilities, lack of awareness on TB, alcohol, smoking, and poor nutrition [6, 13, 17, 22, 23]. Our study findings also point to alcohol, smoking, and low BMI (which reflects poor nutrition) which among the other factors as above could play a significant role in understanding the higher prevalence of TB among the tribal populations in India as a whole.

The age and gender-specific trends of PTB prevalence among the tribal populations reported in our study are comparable to those observed among the general populations [2–4]. PTB prevalence increased with age especially among the elderly age group (>65 years) and was three times higher among males than females. This was similar to other TB prevalence studies from central India among the tribal populations [25, 26]. This higher rate of PTB among the elderly is likely a combination of cumulative years with latent tuberculosis infection (LTBI) and immunosuppressive medication and comorbidities [27]. The higher prevalence in male populationss could also be due to differences in vulnerability and exposure to PTB between men and women [28]. With behaviors such as smoking and alcohol use, coupled with their exposure to dust and air pollution as they travel long distances or migrate to other cities on work [28–30].

Another interesting observation from our study is that the dominant symptom associated with TB among this populations was weight loss followed by night sweat and blood in sputum. Our finding with regard to weight loss is similar to the general population as reported in a study from south India where an association is seen between weight loss and TB (Dhanaraj et al 2015) [31]. This observation is interesting as having a cough is always considered one of the most important symptoms of TB. According to a study by Turner et. al. 2015 [32], there are no studies that have looked at the mechanisms of cough in tuberculosis. There may be coincident factors unrelated to infection that increase the propensity to cough, such as smoking [33, 34] or pre-existing lung disease. This finding is important as it could influence the care seeking behavior of those who present with symptoms of TB.

The study further points out that the risk of acquiring PTB is ten times higher among those who have a history of anti-TB treatment compared to those who do not. This finding stresses the need to capture information on the history of TB, which could be done during screening for symptoms, both while undertaking active case finding or when patients report to health centers for care. This is often not given adequate importance and may pose as a missed opportunity for early diagnosis and treatment initiation when required.

## Conclusion

The overall prevalence of PTB among tribal groups is higher than the general populations. While our study was not powered enough to make conclusion on prevalence of PTB across zones, there is an indication that there seems to be a wide variation of prevalence of PTB across the zones and states in India. This indication calls for more studies to be conducted at the zonal and state level across India as current studies seem to be confined largely to central India. By focusing on just central India the problems associated with TB among the tribal populations have only been explored for the tribal populations in this area. These findings would be of use to the national TB program with efforts underway to eliminate TB by 2025 by assisting the national government to strategically adopt tribal community/site specific TB control intervention programs to reach the unreached tribal populations across India.

## Limitations

One limitation of our study is that we included villages from districts with over 70 percent tribal populations. This has led to the possibility of missing out the PTGs (The primitive tribal groups) which may not have been included in this definition. The study did not include X-ray screening which we tried to overcome by using a correction factor. Another limitation of this study was the restriction of coverage which was due to the high mobility of this populations as many are travel for work outside their districts. Furthermore, our study findings point to a low rate of culture positivity among the smear positive reported cases. This could be attributed to the quality of the sputum considering the challenges of transportation through difficult terrains in these tribal areas.

Although we have provided state wise and zone wise TB prevalence caution should be taken when interpreting the estimates as our study was not powered enough to arrive at justified conclusions.

## Supporting information

S1 Table Univariate analysis for the factors associated with the occurrence of pulmonary tuberculosis. (DOCX)

S1 Map The six geographic zones (A) and states and cluster (i.e. villages) selected randomly from each zone (B) for the tribal prevalence survey in India (The Maps are self-explanatory). (TIFF)

S2 Map Zone (A) and state wise (B) pulmonary tuberculosis prevalence among tribal population. (TIFF)

S1 Fig Relative importance in identifying the presence of tuberculosis. (TIF)

S1 Appendix Dataset to estimate the prevalence of pulmonary tuberculosis among the tribal populations in India. (CSV)

S2 Appendix Data dictionary accompanying the dataset to estimate the prevalence of pulmonary tuberculosis among the tribal populations in India. (TXT)
