# Supplementary material for: Prevalence of pulmonary tuberculosis among the tribal populations in India
Source: PLoS One. 2021 Jun 4;16(6):e0251519. doi: 10.1371/journal.pone.0251519 (PMC8177518; doi:10.1371/journal.pone.0251519)
Supplement: S1 Table — (DOCX) [file pone.0251519.s001.docx]

| **S1 Table: Univariate analysis for the factors associated with the occurrence of pulmonary Tuberculosis** | | | | | | | | | | |
| --- | --- | --- | --- | --- | --- | --- | --- | --- | --- | --- |
|  | **N=74532** | **Smear**  **Positive**  **(n=163)** | **OR**  **(95% CI)** | **Sig.** | **Culture**  **Positive**  **(n=108)** | **OR**  **(95% CI)** | **Sig.** | **Smear/**  **Culture**  **Positive**  **(n=199)** | **OR**  **(95% CI)** | **Sig.** |
|  |  |  |  |  |  |  |  |  |  |  |
| **Age Group** | | | | | | | |  |  |  |
| 15 - 34 | 38390 | 38 (0.1) | 1.00 |  | 23 (0.1) | 1.00 |  | 45 (0.1) | 1.00 |  |
| 35 - 54 | 22973 | 65 (0.3) | 2.86  (1.9 - 4.32) | <0.001 | 50 (0.2) | 3.64  (2.06 - 6.43) | <0.001 | 82 (0.4) | 3.05  (2.08 - 4.48) | <0.001 |
| ≥55 | 13169 | 60 (0.5) | 4.62  (2.99 - 7.14) | <0.001 | 35 (0.3) | 4.45  (2.5 - 7.91) | <0.001 | 72 (0.5) | 4.68  (3.09 - 7.11) | <0.001 |
| **Gender** | | | | | | | |  |  |  |
| Female | 39000 | 53 (0.1) | 1.00 |  | 37 (0.1) | 1.00 |  | 65 (0.2) | 1.00 |  |
| Male | 35532 | 110 (0.3) | 2.28  (1.63 - 3.2) | <0.001 | 71 (0.2) | 2.11  (1.37 - 3.26) | <0.001 | 134 (0.4) | 2.27  (1.65 - 3.12) | <0.001 |
| **Education** | | | | | | | |  |  |  |
| Literate | 42884 | 81 (0.2) | 1.00 |  | 50 (0.1) | 1.00 |  | 93 (0.2) | 1.00 |  |
| Illiterate | 31648 | 82 (0.3) | 1.37  (0.94 - 2) | 0.097 | 58 (0.2) | 1.57  (1.02 - 2.42) | 0.040 | 106 (0.3) | 1.55  (1.09 - 2.19) | 0.014 |
| **Occupation** | | | | | | | |  |  |  |
| Unemployed | 9888 | 5 (0.1) | 1.00 |  | 4 (0.01) | 1.00 |  | 8 (0.1) | 1.00 |  |
| Employed | 64644 | 158 (0.2) | 4.84  (1.74 - 13.46) | 0.003 | 104 (0.2) | 3.98  (0.76 - 20.79) | 0.100 | 191 (0.3) | 3.66  (1.26 - 10.65) | 0.018 |
| **Smoker & Alcohol** | | | | | | | |  |  |  |
| No | 56146 | 103 (0.2) | 1.00 |  | 70 (0.1) | 1.00 |  | 129 (0.2) | 1.00 |  |
| Alcoholic alone | 10657 | 13 (0.1) | 0.66  (0.37 - 1.19) | 0.169 | 6 (0.1) | 0.45  (0.18 - 1.1) | 0.081 | 15 (0.1) | 0.61  (0.35 - 1.06) | 0.080 |
| Smoking Alone | 3353 | 18 (0.5) | 2.94  (1.55 - 5.57) | 0.001 | 14 (0.4) | 3.36  (1.27 - 8.89) | 0.015 | 21 (0.6) | 2.74  (1.35 - 5.57) | 0.006 |
| Both | 4376 | 29 (0.7) | 3.63  (2.37 - 5.57) | <0.001 | 18 (0.4) | 3.31  (1.76 - 6.21) | <0.001 | 34 (0.8) | 3.4  (2.25 - 5.14) | <0.001 |
| **TB History** | | | | | | | |  |  |  |
| No | 73764 | 139 (0.2) | 1.00 |  | 90 (0.1) | 1.00 |  | 171 (0.2) | 1.00 |  |
| Yes | 768 | 24 (3.1) | 17.09  (9.97 - 29.27) | <0.001 | 18 (2.3) | 19.65  (10.55 - 36.6) | <0.001 | 28 (3.6) | 16.28  (9.57 - 27.72) | <0.001 |
| **Drug Use** | | | | | | | |  |  |  |
| No | 69584 | 138 (0.2) | 1.00 |  | 99 (0.1) | 1.00 |  | 172 (0.2) | 1.00 |  |
| Yes | 4948 | 25 (0.5) | 2.56  (1.43 - 4.55) | 0.002 | 9 (0.2) | 1.28  (0.55 - 3) | 0.567 | 27 (0.5) | 2.21  (1.27 - 3.85) | 0.005 |
| **Known Diabetes** | | | | | | | |  |  |  |
| No | 73755 | 160 (0.2) | 1.00 |  | 106 (0.1) | 1.00 |  | 196 (0.3) | 1.00 |  |
| Yes | 777 | 3 (0.4) | 1.78  (0.59 - 5.34) | 0.298 | 2 (0.3) | 1.79  (0.52 - 6.17) | 0.350 | 3 (0.4) | 1.45  (0.49 - 4.36) | 0.499 |
| **Blood Pressure** | | | | | | | |  |  |  |
| No | 72586 | 159 (0.2) | 1.00 |  | 102 (0.1) | 1.00 |  | 190 (0.3) | 1.00 |  |
| Yes | 1946 | 4 (0.2) | 0.94  (0.34 - 2.61) | 0.901 | 6 (0.3) | 2.2  (0.65 - 7.39) | 0.200 | 9 (0.5) | 1.77  (0.72 - 4.37) | 0.213 |
| **HIV** | | | | | | | |  |  |  |
| No | 73629 | 163 (0.2) | Not  Applicable |  | 108 (0.1) | Not  Applicable |  | 199 (0.3) | Not  Applicable |  |
| Yes | 903 | 0 (0) |  |  | 0 (0) |  |  | 0 (0) |  |  |
| **Asthma** | | | | | | | |  |  |  |
| No | 72888 | 147 (0.2) | 1.00 |  | 94 (0.1) | 1.00 |  | 177 (0.2) | 1.00 |  |
| Yes | 1644 | 16 (1) | 4.86  (2.5 - 9.45) | <0.001 | 14 (0.9) | 6.65  (3.36 - 13.17) | <0.001 | 22 (1.3) | 5.57  (3.27 - 9.49) | <0.001 |
| **Malaria** | | | | | | | |  |  |  |
| No | 68621 | 146 (0.2) | 1.00 |  | 98 (0.1) | 1.00 |  | 180 (0.3) | 1.00 |  |
| Yes | 5911 | 17 (0.3) | 1.35  (0.8 - 2.28) | 0.252 | 10 (0.2) | 1.18  (0.6 - 2.35) | 0.624 | 19 (0.3) | 1.23  (0.72 - 2.09) | 0.451 |
| **Body Mass Index** | | | | | | | |  |  |  |
| ≥18.5 | 53961 | 74 (0.1) | 1.00 |  | 52 (0.1) | 1.00 |  | 97 (0.2) | 1.00 |  |
| <18.5 | 20571 | 89 (0.4) | 3.16  (2.07 - 4.83) | <0.001 | 56 (0.3) | 2.83  (1.86 - 4.32) | <0.001 | 102 (0.5) | 2.77  (1.87 - 4.1) | <0.001 |
| OR - Odds Ratio | | | | | | | | | | |
