# Supplementary figures and images for: Prevalence of pulmonary tuberculosis among the tribal populations in India
Source: PLoS One. 2021 Jun 4;16(6):e0251519. doi: 10.1371/journal.pone.0251519 (PMC8177518; doi:10.1371/journal.pone.0251519)

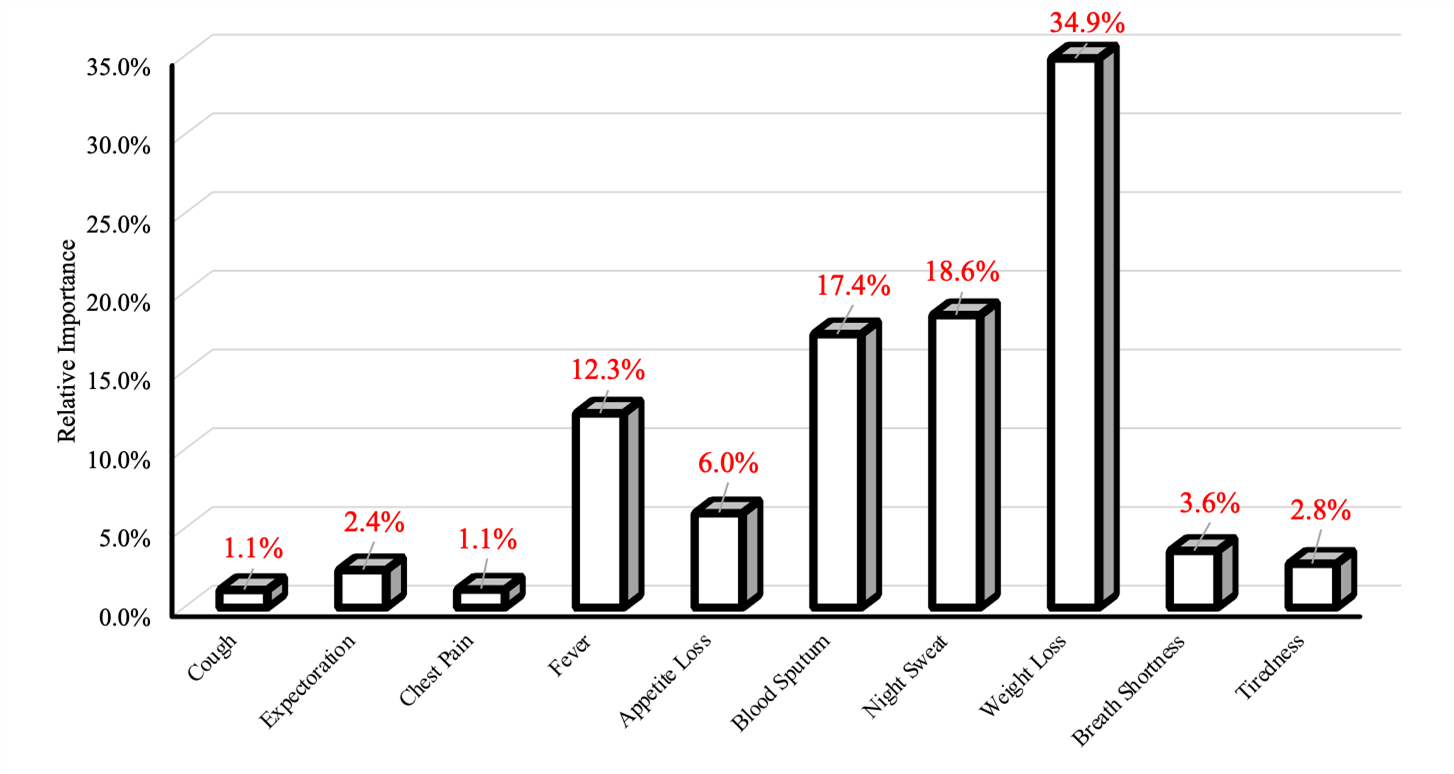

Supplement: S1 Fig — (TIF) [file pone.0251519.s004.tif]
